# Loss of organic cation transporter 3 (Oct3) leads to enhanced proliferation and hepatocarcinogenesis

**DOI:** 10.18632/oncotarget.23372

**Published:** 2017-12-18

**Authors:** Johanna Vollmar, Anja Lautem, Ellen Closs, Detlef Schuppan, Yong Ook Kim, Daniel Grimm, Jens U. Marquardt, Peter Fuchs, Beate K. Straub, Arno Schad, Dirk Gründemann, Jörn M. Schattenberg, Nadine Gehrke, Marcus A. Wörns, Jan Baumgart, Peter R. Galle, Tim Zimmermann

**Affiliations:** ^1^ Department of Internal Medicine, Gastroenterology and Hepatology, University Medical Center, Johannes Gutenberg-University Mainz, Mainz, Germany; ^2^ Department of Hepatobiliary and Transplantation Surgery, University Medical Center, Johannes Gutenberg-University Mainz, Mainz, Germany; ^3^ Department of Pharmacology, University Medical Center, Johannes Gutenberg-University Mainz, Mainz, Germany; ^4^ Institute of Translational Immunology, Fibrosis and Metabolism Center, Johannes Gutenberg-University Mainz, Mainz, Germany; ^5^ Institute of Pathology, University Medical Center, Johannes Gutenberg-University Mainz, Mainz, Germany; ^6^ Department of Pharmacology, University of Cologne, Mainz, Germany; ^7^ Translational Animal Research Center (TARC), Johannes Gutenberg-University Mainz, Mainz, Germany

**Keywords:** organic cation transporter, OCT3 knockout, hepatocarcinogenesis, SLC22A1, SLC22A3

## Abstract

**Background:**

Organic cation transporters (OCT) are responsible for the uptake of a broad spectrum of endogenous and exogenous substrates. Downregulation of OCT is frequently observed in human hepatocellular carcinoma (HCC) and is associated with a poor outcome. The aim of our current study was to elucidate the impact of OCT3 on hepatocarcinogenesis.

**Methods:**

Transcriptional and functional loss of OCT was investigated in primary murine hepatocytes, derived from Oct3-knockout (Oct3^−/−^; *FVB.Slc22a3^tm1Dpb^*) and wildtype (WT) mice. Liver tumors were induced in Oct3^−/−^ and WT mice with Diethylnitrosamine and Phenobarbital over 10 months and characterized macroscopically and microscopically. Key survival pathways were investigated by Western Blot analysis.

**Results:**

Loss of Oct3^−/−^ in primary hepatocytes resulted in significantly reduced OCT activity determined by [^3^H]MPP^+^ uptake *in vivo*. Furthermore, tumor size and quantity were markedly enhanced in Oct3^−/−^ mice (p<0.0001). Oct3^−/−^ tumors showed significant higher proliferation (p<0.0001). *Ki-67* and *Cyclin D* expression were significantly increased in primary Oct3^−/−^ hepatocytes after treatment with the OCT inhibitors quinine or verapamil (p<0.05). Functional inhibition of OCT by quinine resulted in an activation of c-Jun N-terminal kinase (Jnk), especially in Oct3^−/−^ hepatocytes.

**Conclusion:**

Loss of Oct3 leads to enhanced proliferation and hepatocarcinogenesis *in vivo*.

## INTRODUCTION

Hepatocellular carcinoma (HCC) is one of the most frequent human malignancies worldwide [1]. In the majority of cases tumors have a poor prognosis and show a high rate of recurrence [2]. Treatment options, especially with regard to a systemic therapy, are limited [3]. Therefore, new biomarkers and therapeutic options are urgently needed.

Organ cationic transporters (OCT) are membrane transport proteins involved in many metabolic processes and detoxification. Most prominent OCT family members are OCT1 (gene symbol SLC22A1), OCT2 (SLC22A2) and OCT3 (SLC22A3), which have overlapping substrate specificities and tissue expression patterns. In human, OCT1 is mainly expressed in the basolateral membrane of hepatocytes in the liver, OCT2 in the basolateral membrane of epithelial cells in the kidney and genito-urinary system, whereas OCT3 is widely distributed in many tissues including the placenta, heart, liver and skeletal muscle. OCTs functionally substitute each other, ensuring that important metabolic pathways are secured [4, 5].

The transporters became pharmaceutically interesting, because they confer important clinical functions and are responsible for the uptake, intracellular inactivation and biliary or urinary excretion of a broad spectrum of endogenous (e.g. catecholamines) and exogenous substrates (e.g. metformin, betablockers, etc.) as well as anticancer drugs (e.g. platin derivatives or sorafenib) [6–14]. Interestingly, OCT expression has been detected in several cancer cell lines and tumor tissue samples. In this context, we have previously shown a downregulation of OCT1 and OCT3 in human HCC and cholangiocellular carcinoma (CCC), associated with tumor progression and a worse patient survival [15, 16]. However, OCT expression is regulated via complex mechanisms [17, 18]. Although efforts were made, no distinct regulatory pathway has been identified yet. Overexpression of c-Myc, which plays a central role during malignant conversion [19], resulted in an increase of OCT1 protein expression in human breast epithelial cells [20], whereas in HCC-derived tumor cell lines OCT are not relevantly expressed [21].

OCT1 activity was reported to correlate with the sensitivity of tyrosine kinase inhibitors (TKI) in patients with chronic myeloid leukemia (CML) [22–24] and the expression of SLC22A1 variants may affect the response of HCC to sorafenib [25]. In concordance, we recently identified intratumoral OCT1 mRNA expression as a significant positive prognostic factor for patients treated with sorafenib [26]. While these studies clearly demonstrated the impact of OCT1 in HCC, the role of OCT3 is less well understood. To investigate the relevance of OCT3 expression and the effects on hepatocarcinogenesis, liver tumors were chemically induced in Oct3^−/−^ and wild type (WT) mice by administration of diethylnitrosamine (DEN) and phenobarbital. Expression levels of Oct1 were measured in tumors and non-neoplastic tumor surrounding tissue (TST) in Oct3^−/−^ and WT mice, and tumor characteristics including cell proliferation were investigated. Finally, the functional OCT-inhibition was studied in primary murine hepatocytes, derived from Oct3^−/−^ and WT mice.

## RESULTS

### Organic cation transport is decreased in Oct3^−/−^ hepatocytes

**Figure 1:**
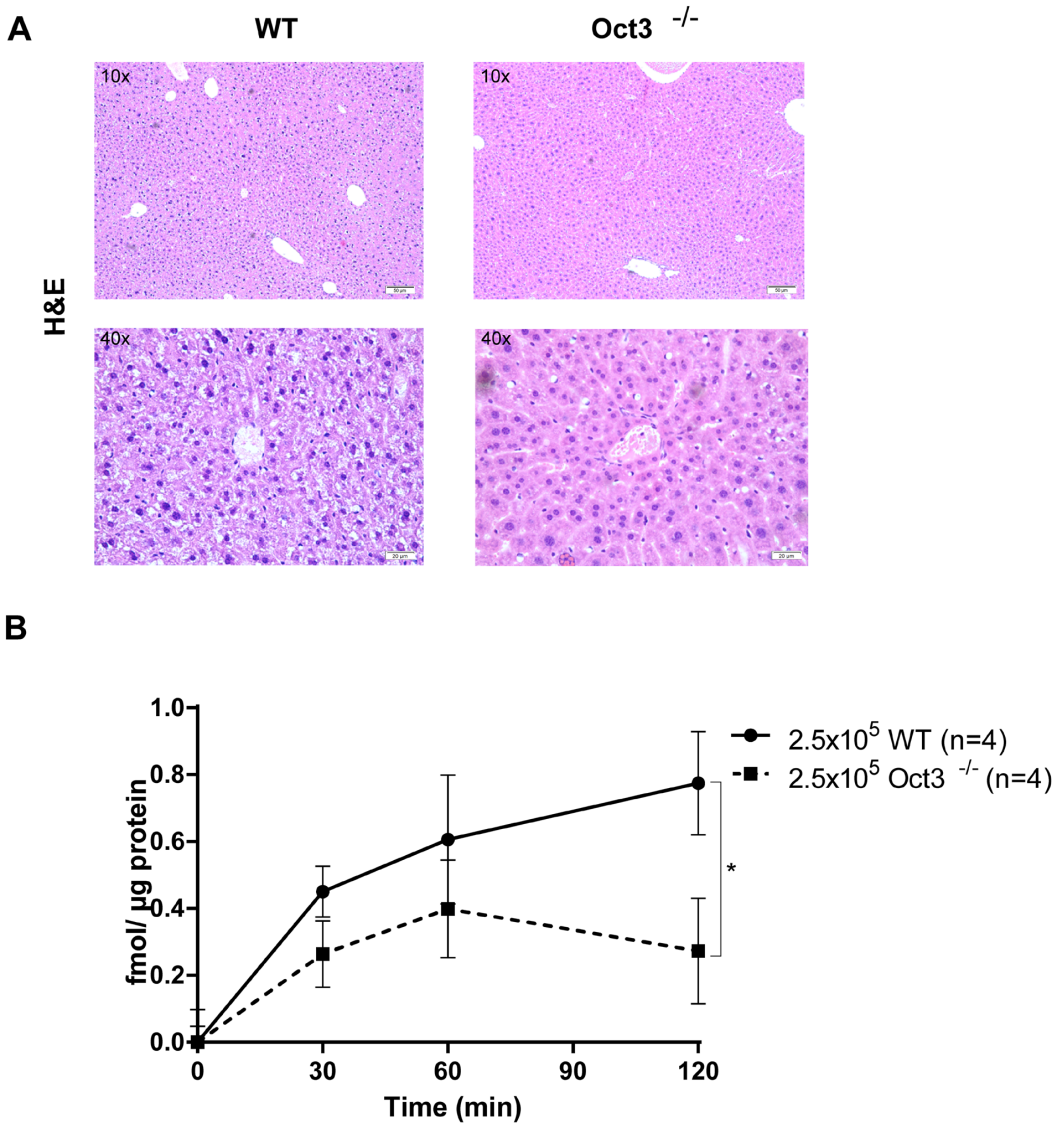
**(A)** H&E staining of untreated WT and Oct3^−/−^ liver tissue. Representative histological images are shown. **(B)** [^3^H]MPP^+^ uptake in primary hepatocytes: 2.5×10^5^ WT or Oct3^−/−^ hepatocytes (n=4) were incubated with [^3^H]MPP^+^, an Oct substrate, for 0, 30, 60 and 120 seconds and [^3^H]MPP^+^ uptake was quantified as fmol [^3^H]MPP^+^/ μg protein.

The liver of Oct3^−/−^ mice appeared histologically normal and no distinct morphological difference could be detected between Oct3^−/−^ mice and WT littermates at the age of 10 months (Figure 1A).

Consistently, no significant differences were found for activity of serum alanine transaminase (ALT) and aspartate transaminase (AST), alkaline phosphatase (AP), bilirubin and lactate dehydrogenase (LDH) between Oct3^−/−^ and WT mice (Supplementary Table 1).

To test the consequences of Oct3 deficiency on organic cation transport, primary Oct3^−/−^ and WT hepatocytes were incubated with tritium labeled 1-methyl-4-phenylpyridinium ([^3^H]MPP^+^), as a standard substrate, for 0, 30, 60 and 120 seconds and [^3^H]MPP^+^ uptake was quantified. As expected, [^3^H]MPP^+^ transport was significantly decreased in Oct3^−/−^ hepatocytes with a mean uptake of 0.3 fmol [^3^H]MPP^+^/μg protein after 120 seconds in comparison to WT hepatocytes with a mean uptake of 0.8 fmol [^3^H]MPP^+^/μg protein (p=0.012) (Figure 1B).

### Increased tumorigenesis in Oct3^−/−^ mice after 10 months of DEN/Phenobarbital treatment

**Figure 2:**
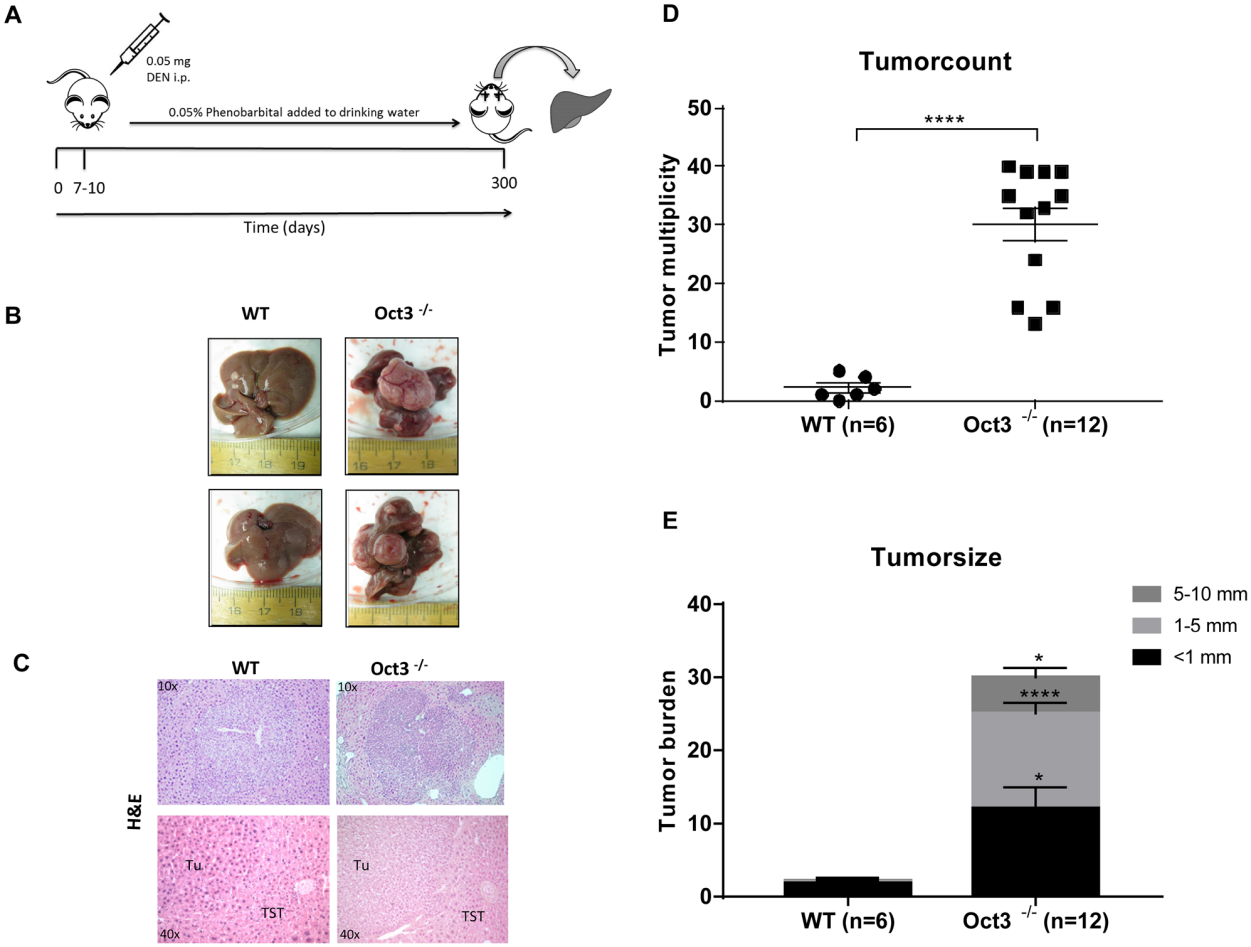
Liver tumor development **(A)** A single DEN (0.05 mg per mouse) i.p. injection was performed between days 7 and 10 postpartum. Phenobarbital (0.05%) was continuously added to drinking water. Mice were sacrificed at 10 months and liver collected for histology and protein analysis. **(B)** Exemplarily macroscopic and **(C)** Microscopic (H&E) tumor appearance after 10 months of DEN-initiated and Phenobarbital-promoted tumorigenesis in WT and Oct3^−/−^ mice. **(D)** Quantitative liver tumor development after induction with DEN/Phenobarbital over 10 months: tumor nodules on the liver surface of all lobes were counted in 12 Oct3^−/−^ and 6 WT mice. **(E)** Tumorsize after induction with DEN/Phenobarbital over 10 months: the size of tumor nodules on the liver surface of all lobes was measured in 12 Oct3^−/−^ and 6 WT mice and categorized as <1mm, 1-2 mm and 5-10 mm. Subsequently, the tumor burden of each category was counted.

To evaluate the impact of Oct3 deficiency for hepatocarcinogenesis, liver tumors were induced by DEN/Phenobarbital and tumor growth was compared to WT littermates after a total of 10 months (Figure 2A). Loss of Oct3 led to a significant increase in liver tumor formation (Figure 2B-2D). Comparing the number of liver tumors of Oct3^−/−^ mice to that of WT mice, the knockout mice demonstrated a significantly increased incidence of developing liver tumors (p<0.0001). In mean, Oct3^−/−^ mice developed 30.08 ± 2.92 tumor nodules in comparison to 2.17 ± 0.79 nodules in WT mice (Figure 2D). Consistently, a significant increased tumor volume per nodule was observed in Oct3^−/−^ in comparison to WT mice: on average, the majority of tumors of the Oct3^−/−^ livers measured 1-5 mm in diameter, whereas the WT tumors, only showed diameters below 1 mm (p<0.0001) (Figure 2E).

### Characterization of Oct3^−/−^ liver tumors reveals enhanced proliferation

**Figure 3:**
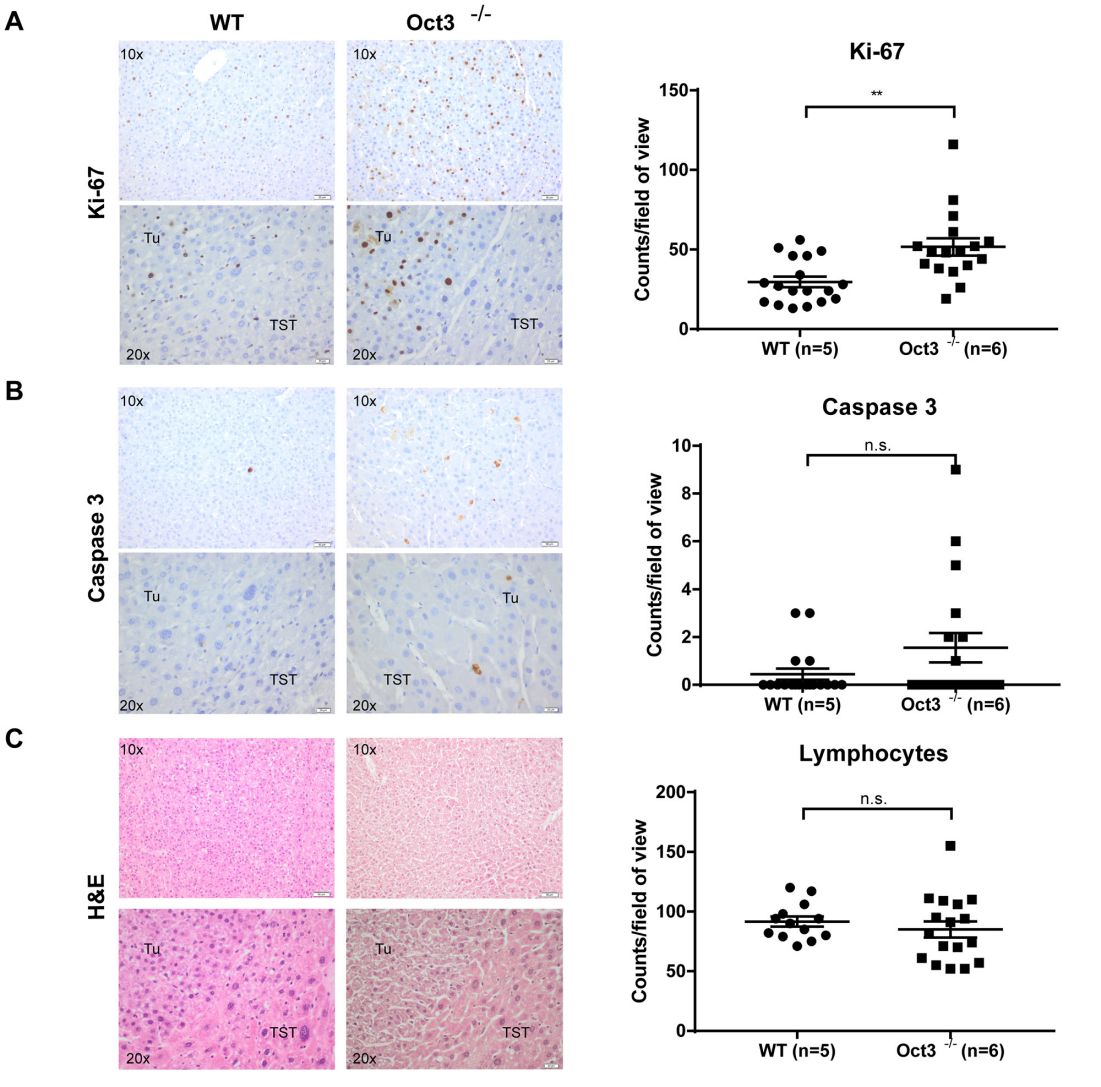
Tumor characterization As a measurement of proliferation and apoptosis the total number of **(A)** Ki-67 and **(B)** Caspase 3 positive cells/field of view was counted in immunohistochemistry. **(C)** For evaluation of inflammation, the total number of lymphocytes/field of view was counted in H&E staining. Tumors of 5 WT and 6 Oct3^−/−^ mice were evaluated and three fields of view were recorded per mouse. Representative histological images are shown.

To further characterize the oncogenic characteristics of the liver tumors in Oct3^−/−^ mice, immunohistochemistry against Ki-67, Caspase 3 and lymphocyte infiltration was performed. In concordance with the observed increase in tumor number and volume, an increased cell proliferation was detected in tumors of Oct3-deficient animals (n=6) compared to WT mice (n=5). Regarding Ki-67 positive cells, significant higher counts were detected in Oct3^−/−^ derived liver tumors in comparison to tumors of WT mice (51.65 ± 5.41 vs. 29.61 ± 3.3 Ki-67 positive cells per field of view; p=0.0013) (Figure 3A). Tumors of Oct3^−/−^ mice exhibited a non-significant trend towards increased apoptosis in comparison to tumors of WT mice (0.44 ± 0.23 vs. 1.556 ± 0.62 caspase-3-positive cells per field of view; p=0.1) (Figure 3B). Inflammation in Oct3^−/−^ and WT derived tumors was comparable (91.62 ± 4.27 vs. 85 ± 6.76 lymphocytes per field of view; p=0.45) as assessed by H&E staining (Figure 3C). No significant difference was found in the serum liver values (ALT, AST, alkaline phosphatase, total bilirubin and lactate dehydrogenase) between Oct3^−/−^ and WT mice (Supplementary Table 1).

### Oct1 mRNA expression is increased in non-neoplastic tumor surrounding tissue of Oct3^−/−^ vs. WT mice in the DEN/Phenobarbital model and is downregulated in tumor tissue

**Figure 4:**
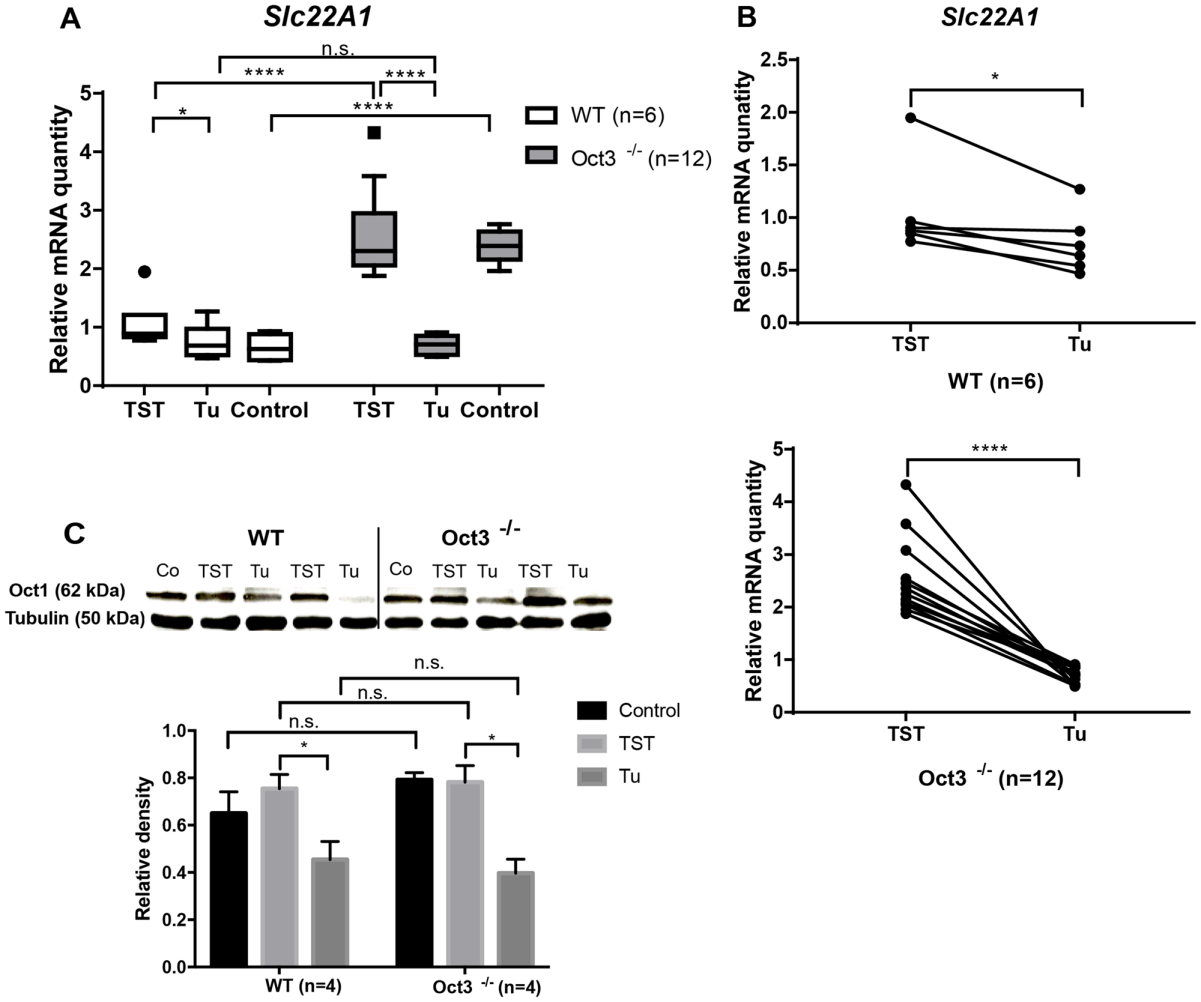
Oct1 expression **(A)** *Slc22A1* mRNA expression in TST, tumor and control tissue was determined by qPCR in 12 OCT3^−/−^ and 6 WT mice treated with DEN/ Phenobarbital for 10 months. The relative expression levels of *Slc22A1* were calculated by normalization to GAPDH gene expression. **(B)** Individual *Slc22A1* mRNA expression pattern in each mouse (HCC and according TST). **(C)** Western blot analysis of two representative Oct3^−/−^ and WT mice and densitometric analysis in tumors (Tu) and corresponding TST (n=4). Normal liver tissue derived from 10 months old untreated Oct3^−/−^ and WT mice served as control (Co) and Tubulin as loading control (Oct1: 62 kDa, Tubulin: 50 kDa).

In order to determine Oct1 expression, *Slc22A1* mRNA expression was analyzed in tumor and tumor-surrounding liver tissue of Oct3^−/−^ and WT mice and untreated controls by qPCR. In accordance with the findings in human HCCs [15], *Slc22A1* mRNA expression was downregulated in tumor compared to tumor-surrounding tissue (Figure 4A). *Slc22A1* mRNA was 1.8-fold (+/− 0.2-fold) lower in tumor tissue of Oct3^−/−^ mice, and 0.3-fold (+/− 0.09-fold) lower in tumors of WT mice, compared to TST. Remarkably, *Slc22A1* mRNA expression was significantly higher in untreated controls and TST of Oct3^−/−^ mice in comparison to WT mice (p<0.001), but no difference was detected in tumor tissue (p=0.57). Individual expression patterns in liver tumors and corresponding TST are demonstrated in Figure 4B.

To examine if Oct3 deficiency also affects protein expression of Oct1 in tumors and corresponding TST in Oct3^−/−^ and WT mice, we performed Western blot analysis. Immunoblotting revealed that the downregulation of *Slc22A1* mRNA expression in tumor tissue in comparison to TST as measured in the qPCR (Figure 4A, 4B) correlates with the corresponding protein levels in liver tumors and TST in Oct3^−/−^ and WT mice (Figure 4C).

### Functional inhibition of OCT induces Slc22A1 and Slc22A3 mRNA expression

**Figure 5:**
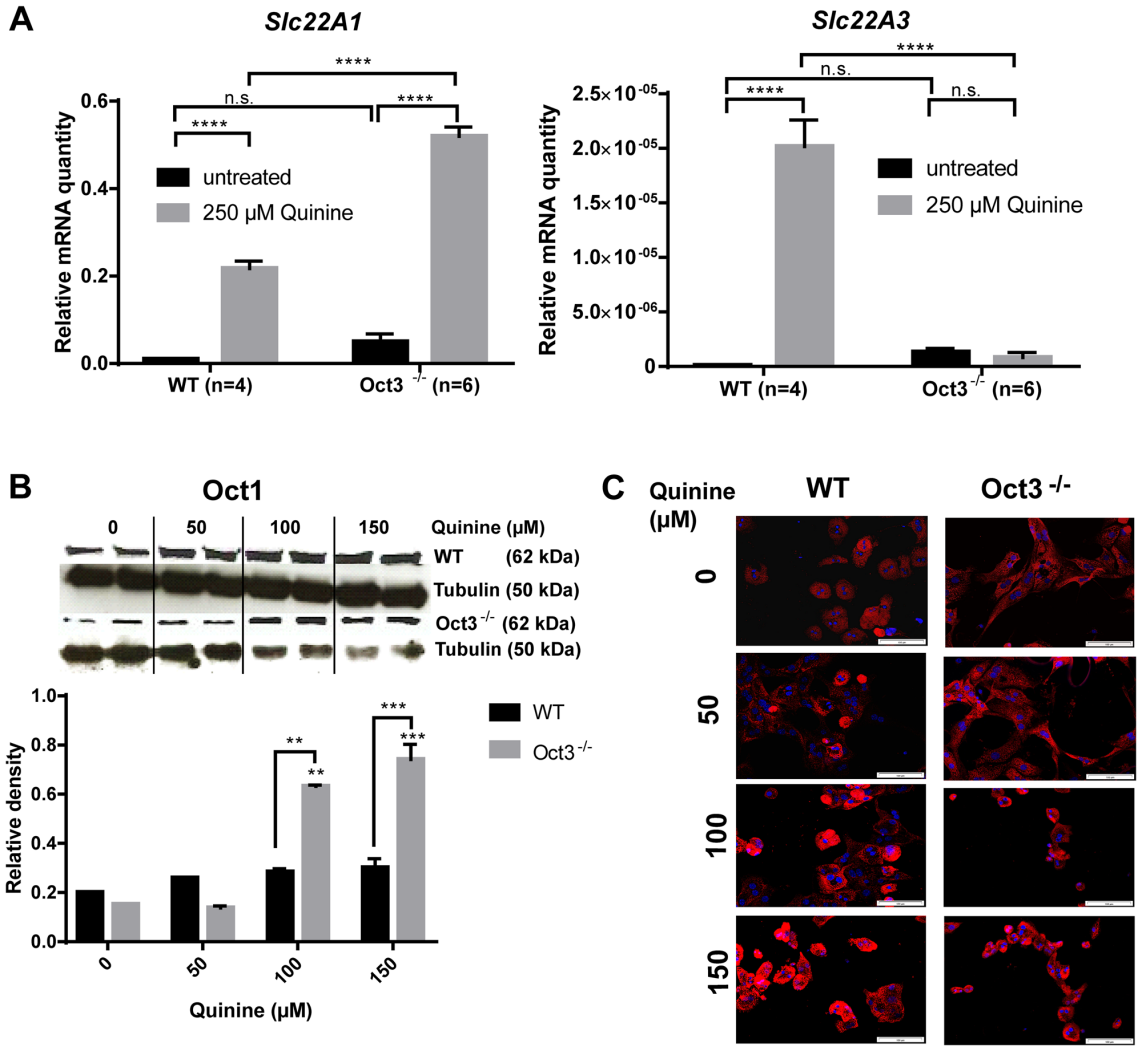
Oct regulation **(A)** *Slc22A1* and *Slc22A3* mRNA expression were quantified by qPCR in primary WT (n=4) and Oct3^−/−^ (n=6) hepatocytes after treatment with 250 μM quinine. The relative expression levels of *Slc22A1* were calculated by normalization to GAPDH gene expression. **(B)** Oct1 protein expression in primary murine Oct3^−/−^ and WT hepatocytes treated with escalating quinine doses (0, 50, 100 and 150 μM) was determined by western blot analysis. Representative western blots and densitometric analysis are shown. **(C)** Corresponding Oct1 immunofluorescence of primary murine hepatocytes treated with quinine (0, 50, 100 and 150 μM).

The underlying regulatory mechanisms cannot be easily studied *in vivo* and there are no Oct1^−/−^/3^−/−^ mice available. As OCTs are not relevantly expressed in HCC-derived tumor cell lines [21] experiments with primary hepatocytes isolated from Oct3^−/−^ and WT mice were performed. *Slc22A1* and *Slc22A3* mRNA expression were measured by qPCR and Oct1 protein expression was determined by Western Blot analysis and immunofluorescence. *Slc22A1* mRNA expression was significantly upregulated in primary WT and Oct3^−/−^ hepatocytes after treatment with quinine (p<0.0001) (Figure 5A), whereby the upregulation of *Slc22A1* mRNA expression was significantly higher in hepatocytes derived from Oct3^−/−^ mice than WT hepatocytes (p<0.0001). *Slc22A3* mRNA expression was significantly upregulated in primary WT (p<0.0001) (Figure 5A). In concordance, Oct1 protein expression increased with escalating doses of quinine (0, 50, 100 and 150 μM) in primary murine hepatocytes (Figure 5B, 5C). These data suggest that *Slc22A1* and *Slc22A3* mRNA expression are induced by a functional inhibition of the transporter.

### Inhibition of transporter function results in increased proliferation

**Figure 6:**
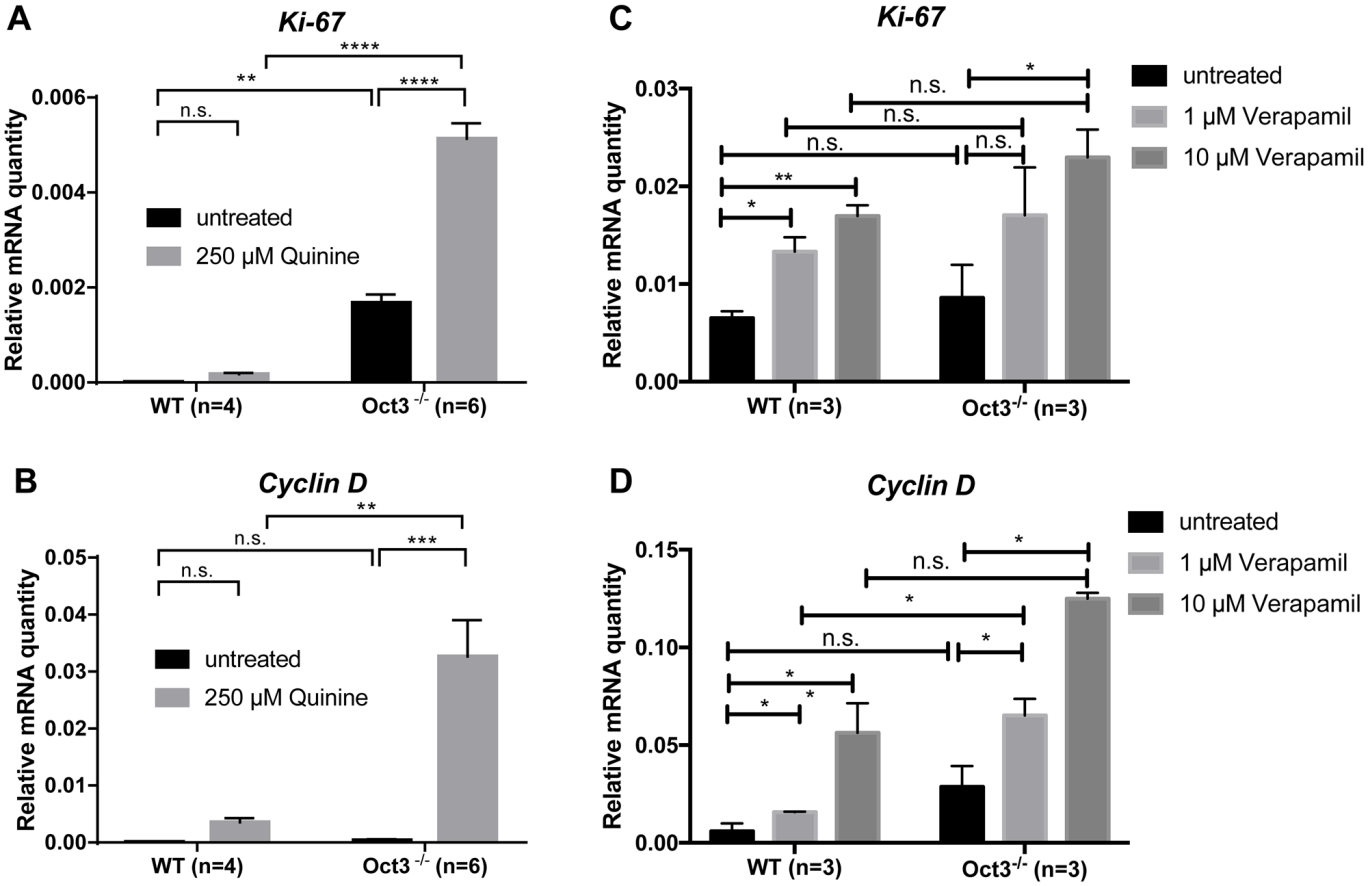
Loss of Oct function results in enhanced proliferation **(A)** *Ki-67* and **(B)** *Cyclin D* mRNA expression were determined by qPCR in primary Oct3^−/−^ (n=6) and WT (n=4) hepatocytes after a 24 hour 250 μM quinine treatment. **(C)** *Ki-67* and **(D)** *Cyclin D* mRNA expression were determined by qPCR in primary Oct3^−/−^ (n=3) and WT (n=3) hepatocytes after 24 hours treatment with escalating doses of verapamil (1 μM and 10 μM). The relative expression levels were calculated by normalization to GAPDH gene expression.

To examine whether suppression of transporter function also influences cell proliferation and cell cycle, *Ki-67* and *Cyclin D* mRNA expression was determined by qPCR in primary hepatocytes derived from Oct3^−/−^ and WT mice after a 24 hours treatment with the Oct inhibitors quinine or verapamil (Figure 6).

*Ki-67* and *Cyclin D* mRNA expression were significantly upregulated in primary Oct3^−/−^ hepatocytes after treatment with quinine (p<0.001) (Figure 6A, 6B) or verapamil (p<0.05) (Figure 6C, 6D). This effect was dose dependent (Figure 6C, 6D).

Primary Oct3^−/−^ hepatocytes treated with quinine, showed a significant higher *Ki-67* and *Cyclin D* mRNA expression than WT hepatocytes (p<0.01) (Figure 6A, 6B), suggesting that transcriptional loss of Oct3 results in increased proliferation.

### Loss of OCT function leads to activation of the JNK pathway

**Figure 7:**
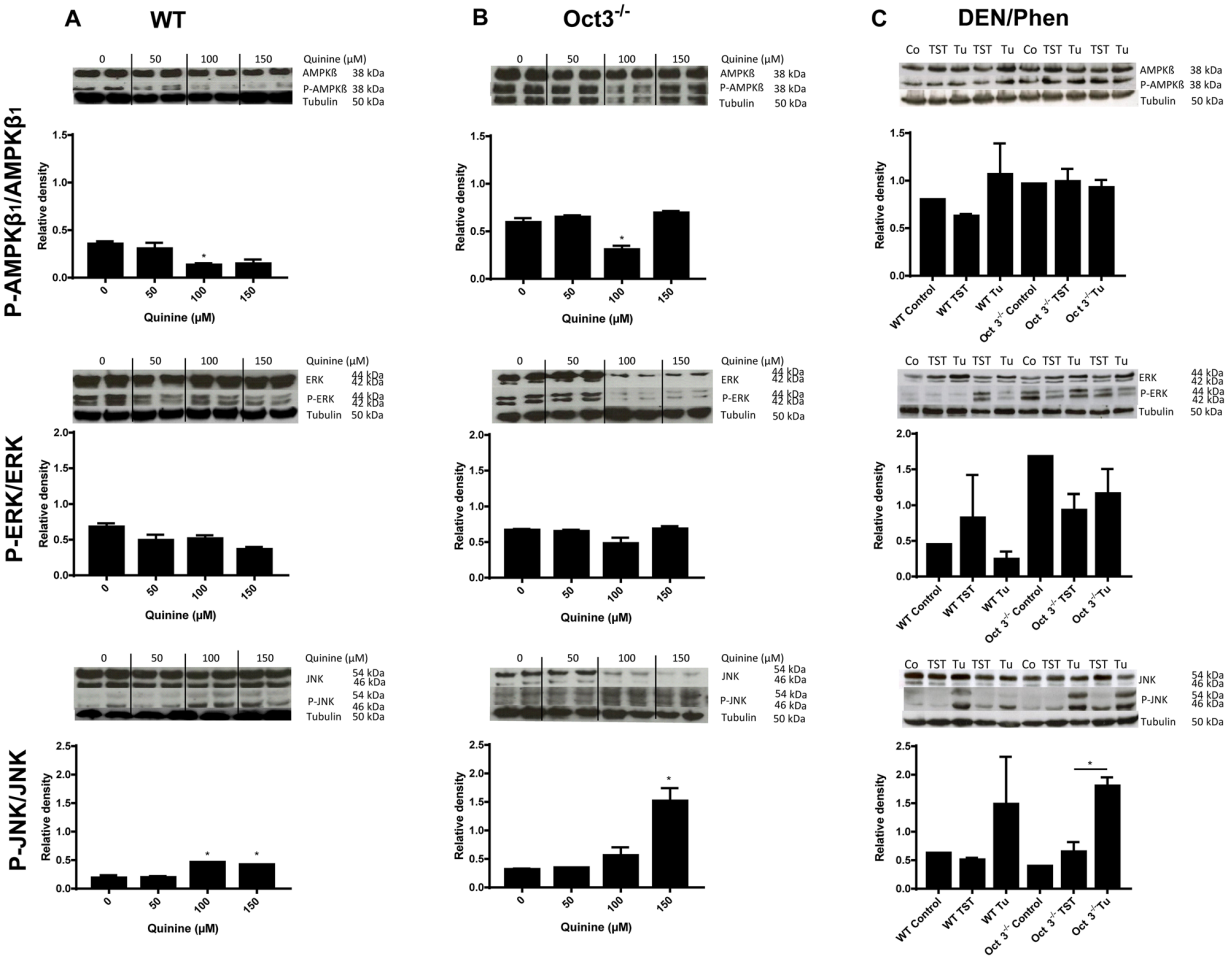
Pathway analysis Protein levels of key enzymes of the Ras-Raf-Mek-Erk, Jnk and mTOR pathways (Ampkβ, P-Ampkβ, Erk, P-Erk, Jnk and P-Jnk) were determined by western blot analysis in primary murine hepatocytes treated with different quinine concentrations (0, 50, 100 and 150 μM) **(A, B)** and in tumors (Tu) vs. TST of Oct3^−/−^ and WT mice after 10 months of DEN/Phenobarbital (Phen) treatment **(C)**. Representative western blots are shown and densitometric quantification was expressed as ratio of phosphorylated protein to total protein. Controls were untreated WT and Oct3^−/−^ mice.

To elucidate the underlying mechanisms involved in increased proliferation and tumorigenesis in Oct3^−/−^ mice, protein levels of key enzymes of the Ras-Raf-Mek-Erk, Jnk and mTOR pathways (Akt, phosphorylated (P-) Akt, Mek, P-Mek, Ampkβ, P-Ampkβ, Ampkα, P-Ampkα, Erk, P-Erk, Jnk and P-Jnk) were determined in primary murine hepatocytes treated with different quinine concentrations (0, 50, 100 and 150 μM) and in tumors and TST of Oct3^−/−^ and WT mice by western blot analysis. P-Ampkβ_1_ was significantly downregulated in WT and Oct3^−/−^ hepatocytes with 100 μM quinine (p<0.05), while P-Erk was not significantly altered with quinine treatment (p>0.05) (Figure 7A, 7B). P-Jnk was upregulated in both quinine treated primary murine hepatocytes (p<0.05) (Figure 7A, 7B) and in tumors of Oct3^−/−^ mice (p<0.05) (Figure 7C). P-Ampkα and Akt was not significantly changed in primary murine hepatocytes with quinine treatment, TST and tumors of WT and Oct3^−/−^ mice (data not shown).

## DISCUSSION

Genetic variants in SLC22A3 were reported to contribute to the risk of distal colon cancer in an Asian population [27]. We have recently shown that a downregulation of OCT1 and OCT3 in human HCC and CCC is associated with tumor progression and a worse patient survival [15, 16]. Downregulation of SLC22A1 mRNA expression in primary human HCC is in line with published microarray findings by Park et al., where SLC22A1 showed a significantly reduced expression in HCC [28].

Functional and transcriptional loss of OCT, e.g. by aberrant OCT1 variants, could be responsible for a reduced or lacking response towards sorafenib and platin derivatives [25, 26]. Therefore, a reliable mouse model is urgently needed to study the role of OCT function in hepatocarcinogenesis, determine the underlying mechanisms, identify new therapeutic targets and develop novel antitumor strategies. For the first time, we present a phenotype of Oct3^−/−^ mice with an impaired substrate uptake of Oct3^−/^ *in vivo*. These findings are relevant as it was described that OCTs functionally substitute each other in order to compensate a loss of function [4, 5]. Our experiments demonstrate that this mechanism is insufficient in Oct3^−/−^ mice: [^3^H]MPP^+^ transport as a substrate for Oct1 and Oct3 was significantly decreased in Oct3^−/−^ hepatocytes, indicating that Oct3 transporter function was not completely substituted in Oct3^−/−^ mice. Furthermore, these mice developed more and larger liver tumors (p<0.0001) under treatment with DEN and Phenobarbital over 10 months. Liver tumors derived from Oct3^−/−^ mice revealed significantly more proliferation (p<0.01) than tumors derived from livers of WT mice. These findings implicate that Oct3^−/−^ mice are more susceptible for liver tumor development due to enhanced proliferation and match the results in human HCC that the downregulation of OCT expression is associated with advanced tumor stages [15]. To date no data exist on the underlying mechanisms of Oct regulation in murine HCC.

Interestingly, the transporters could be directly associated with mechanisms of tumor development by influence of environmental factors and carcinogens. In HepG2 cells expression of SLC22A3 correlates with the carcinogenic potency after treatment with polycyclic aromatic hydrocarbons (PAH), which are known to be carcinogens [29]. Although, it is technically not possible to verify the intratumoral functionality of the single transporters, DEN is not a substrate for OCTs because of a pKA-value of 3.5, since under physiological conditions (pH=7.4) all molecules are uncharged. A positive charge is strictly necessary for transported substrates.

We did not use tumor cell lines to examine endogenous OCT expression and functionality because there is a strong downregulation in all cancer cell lines, which makes these models highly artificial and defective [21]. Furthermore, cancer cell lines have been extensively studied, showing very different results compared to primary hepatocytes [30]. In primary WT and Oct3^−/−^ hepatocytes *Slc22A1* mRNA expression was significantly upregulated after 24 hours of quinine treatment *in vivo* (p<0.05). *Ki-67* and *Cyclin D* mRNA expression were upregulated in primary Oct3^−/−^ hepatocytes (p<0.001) after 24 hours of quinine or verapamil treatment indicating enhanced proliferation with loss of Oct3. In contrast to our findings, Zwart et al reported that Oct3^−/−^ mice show no overt neural or physiological dysfunction [5]. However, quinine can be hypothesized to have Oct1/3-independent effects. Therefore, we validated our findings with the quinine-independent OCT inhibitor verapamil in primary hepatocytes. There is no selective inhibitor for Oct1 or Oct3. All inhibitors known, block both transporters. In addition, the dose-dependence of verapamil towards Ki-67 and Cyclin D expression was investigated. Cell proliferation showed a corresponding upregulation towards escalating doses of verapamil.

Analyzing the protein expression of regulatory key enzymes of the Ras-Raf-Mek-Erk, Jnk and mTOR signaling pathways in TST and tumors of WT and Oct3^−/−^ mice, we found differences in all investigated regulatory pathways (Ras-Raf-MEK-ERK, JNK and mTOR), but no distinct regulatory pathway is known. Chen et al. describe changes in cell metabolism with loss of OCT1 [18] and Fu et al. showed complex OCT3 imprinting mechanisms involved in metastasis in familial esophageal cancer [31]. There were minor differences in the expression levels of Erk and Ampkβ_1_ but we found no clear effect of the OCT inhibitor quinine on Erk- and Ampk-signaling in Oct3^−/−^ and WT hepatocytes. In contrast, an activation of the Jnk pathway was shown in primary hepatocytes of WT and Oct3^−/−^ mice by quinine treatment. Jnk was more activated by quinine (150 μM) in Oct3^−/−^ hepatocytes than in wild-type counterparts (Figure 7B). C-Jun N-terminal kinases (JNKs) are involved in the cellular stress response and malignant transformation [32–34]. Chang et al. demonstrated that higher JNK1 activation was associated both with a poorer prognosis of the patients and with the overexpression of several hepatic stem cell or progenitor cell markers, such as EpCAM, CD24, CD133, KRT19, and AFP [35]. In mouse HCC models, genetic disruption of the Jnk1 locus substantially reduced the number and size of HCCs that were induced by DEN [36].

In this paper we investigated the impact of loss of Oct3 on hepatocarcinogenesis and tumor progression. Tumor initiation was not part of this work, but mutation in OCT gene was associated with enhanced incidence of colorectal cancer in an Asian population [27].

In summary, loss of Oct3 leads to enhanced proliferation and hepatocarcinogenesis. Therefore, these transporters could become novel players in the development of therapeutic and diagnostic tools for HCC. A therapeutic approach with re-expression of Oct3 in HCC may reveal that Oct3 could function as a target in HCC. Further efforts are necessary to elucidate the complex mechanisms of OCT regulation.

## MATERIALS AND METHODS

### Transport experiments

Primary hepatocytes were isolated from Oct3^−/−^ and WT mice and cultured in collagen-coated 24-well culture plates (2.5×10^5^/ml) as described previously [37]. Cells were incubated with 0.1 μM 1-Methyl-4-phenylpyridinium (MPP-Iodid by Sigma-Aldrich, St. Louis, MO, USA, D048) for 5 minutes. Afterwards 2 μCi/ml [3H]MPP^+^ (specific activity 80 Ci/mmol, concentration 1 mCi/ml in ethanol, by Hartmann) was added and cells were incubated for 0, 30, 60 or 120 seconds according to manufacturer`s instruction. In order to stop transport activity cells were washed 3 times with cold Locke’s solution (LS; composition: 154 mmol/l NaCl; 5.6 mmol/l KCl; 2 mmol/l CaCl_2_; 1 mmol/l MgCl_2_; 10 mmol/l HEPES; 3.6 mmol/l NaHCO_3_; 5.6 mmol/l glucose). After lysis with 0.5 M NaOH and neutralization with 0.5 M HCl, cell lysates were mixed with scintillation fluid. The [^3^H]MPP^+^ content was determined in a scintillation counter. Total protein was measured in cell lysates as described [38]. MPP^+^ transport values were expressed as fmol [^3^H]MPP^+^/μg protein.

### Animals

Animal care and animal procedures were in accordance with the governmental and institutional guidelines. Animal experiments were performed in accordance with the European Council Directive of November 24, 1986 (86/609/EEC), and were approved by the state animal care commission. Oct3-knockout (*FVB.Slc22a3^tm1Dpb^*, Oct3^−/−^) mice [5] and their wildtype (WT) littermates were used in this study.

### Initiation of liver tumor development

HCC development in mice was achieved by treatment with DEN and Phenobarbital as described [39]. Hepatocarcinogenesis was induced by a single intraperitoneal (i.p.) injection of diethylnitrosamine (DEN) at a fix dose of 0.05mg in male Oct3^−/−^ mice and WT littermates 7-10 days postpartum followed by continuous treatment with phenobarbital added to drinking water (0.05%). Mice were sacrificed at 10 months and their livers harvested. 10 months old untreated Oct3^−/−^ and WT mice served as control.

### Macroscopical and microscopical analysis

Livers were assessed visually and tumor nodules on the liver surface of all lobes were counted and their size measured. To investigate liver structure and tumor histology, formalin-fixed and paraffin-embedded liver sections (5 μm thick) were stained with Hematoxylin and Eosin (H&E) using standard protocols. For evaluation of inflammation, the total number of lymphocytes/field of view was counted.

### Immunohistochemistry

Immunohistochemistry was performed on frozen liver sections (6 μm thick). Ki-67 and Caspase 3 staining was performed on acetone-fixed sections by using 1:50 rat-anti-mouse Ki-67 (eBioscience, Inc, San Diego, CA, USA) and rabbit-anti-mouse Caspase 3 (Cloud Clone Corp, Houston, TX, USA) as primary antibodies. Signal detection was performed using ‘Vectastain ABC Kit’ (Vector Laboratories, Burlingame, USA) and ‘Fuchsin Substrate-Chromogen System’ (DakoCytomation, Glostrup, Denmark). As a measurement of proliferation and apoptosis the total number of Ki-67 and Caspase 3 positive cells/field of view was counted.

### Immunofluorescence

Primary murine hepatocytes were incubated with rabbit-polyclonal-anti SLC22A1 (GeneTex International Corporation, Irvine, CA, USA) as primary antibody after preincubation with hydrogen peroxide for blocking of endogenous peroxidase. Endogenous biotin was blocked with the Avidin-Biotin Blocking kit (Vector Laboratories, Burlingame, CA, USA) and contaminating proteins were inhibited by ROTI®-Immunoblock solution (ROTH, Karlsuhe, Germany). After incubation with the secondary antibody (goat anti-rabbit IgG-Biotin, 1:1000; DAKO Cytomation, Hamburg, Germany) the TSA™ Cyanine system (Perkin Elmer, Waltham, MA, USA) was added. For negative control the primary antibody was omitted. The images were evaluated under a fluorescence microscope (Olympus BX51, Olympus U-RFL-T).

### RNA isolation and real-time RT-PCR analysis

Total RNA was extracted from liver tissue using the High Pure RNA Tissue Kit (Roche, Mannheim, Germany) and cDNA synthesis was performed using the iScript cDNA Synthesis kit (Biorad, München, Germany) according to the manufacturer’s recommendations. Quantitative analysis of Oct1 *(Slc22A1)* transcripts was performed by quantitative real-time reverse transcriptase (RT-) polymerase chain reaction (qPCR). The Quantitect SYBR Green PCR Kit (Qiagen, Hilden, Germany) and validated primers of a Quantitect Primer Assay with the primer sets Mm_SLC22A1_2_SG (OCT1; 84 bp fragment) and Mm_GAPDH_3_SG (GAPDH; 144 bp fragment) (Qiagen, Hilden, Germany) were used according to the manufacturer’s instructions. Primers additionally applied are listed in the supplementary data (Supplementary Table 2). For the amplification, an initial denaturation at 95°C for 15 min, followed by 15 s at 94°C, 30s at 55°C and 30s at 72°C for 40 cycles was used. Samples were run on a LightCycler® 480 real-time PCR system (Roche, Mannheim, Germany). The relative expression levels of Oct1 (*Slc22A1*) in HCC and TST were calculated by normalization to GAPDH gene expression using the LightCycler® 480 software Release 1.5.0.

### Western blot analysis

Total protein extracts were prepared in sample buffer pH 8.0 containing 20 mM Tris, 5 mM EDTA, 0.5% TritonX-100 and complete Mini, EDTA-free protease inhibitors (1:25; Roche Diagnostics, Germany). For Western blot analysis 60 μg total protein was separated by a 12% SDS-PAGE gel. The gel was transferred onto a nitrocellulose transfer membrane (OPTITRAN BA-S85/Whatman) following separation. Rabbit-anti-OCT1 monoclonal antibody (1:1000; Genway Biotech, Inc, San Diego, CA, USA) or rabbit-anti-α-tubulin polyclonal antiserum (1:1000; Abcam, Cambridge, UK) were used as primary antibody. Antibodies additionally applied are listed in the supplement (Supplementary Table 3). Horseradish peroxidase (HRP)-conjugated anti-rabbit IgG (Santa Cruz Biotechnology, Inc, Dallas, TX, USA) was used as secondary antibody at 1:10000 dilution. Protein bands were visualized using Western Lightning® Plus-ECL enhanced chemiluminescent substrate (Perkin Elmer, Waltham, MA, USA). Densitometry was performed with Image J, an open source image processing program designed for scientific multidimensional images (www.imagej.net).

### OCT inhibition

For functional inhibition of the transporters primary murine hepatocytes were treated with different doses of the non-selective OCT inhibitors quinine (Sigma-Aldrich, St. Louis, MO, USA) [40, 41] or verapamil (Sigma-Aldrich, St. Louis, MO, USA) for 24 hours. *Slc22a1*, *Ki-67* and *Cyclin D* expression were measured by qPCR.

### Statistical analysis

Data management and statistical analysis were performed with Prism version 7.0 (GraphPad Software, Inc., San Diego, CA). Results are expressed as means ± SEM and represent data from a minimum of three independent experiments assessed in triplicate. As sample numbers were small, t-distribution was assumed. Where two groups were compared, Student’s *t* test was used. Data with more than two groups were analyzed by ANOVA and Dunnett`s multiple comparisons test. P < 0.05 was considered statistically significant.

## CONCLUSION

A phenotype of Oct3^−/−^ mice with increased hepatocarcinogenesis was identified and characterized. Oct3^−/−^ primary hepatocytes showed a reduced substrate uptake. Functional inhibition of Oct3^−/−^ primary hepatocytes induced proliferation and Jnk-activation.

## SUPPLEMENTARY MATERIALS TABLES



## Abbreviations

OCT1/2/3: organic cation transporter 1/2/3 (gene symbol: SLC22A1, SLC22A2, SLC22A3)
HCC: hepatocellular carcinoma
Oct3^−/−^: OCT3-knockout, FVB.Slc22a3^tm1Dpb^
WT: wildtype
DEN: diethylnitrosamine
TST: tumor surrounding tissue
qPCR: quantitative real-time reverse transcriptase polymerase chain reaction
TKI: tyrosine kinase inhibitors
CML: chronic myeloid leukemia
MPP: 1-Methyl-4-phenylpyridinium
Jnk: c-Jun N-terminal kinase
H&E: Hematoxylin and Eosin
HRP: horseradish peroxidase
ALT: serum alanine transaminase
AST: aspartate transaminase
AP: alkaline phosphatase
LDH: lactate dehydrogenase
P-: phosphorylated
Phen: Phenobarbital
Tu: tumor

## References

[1] El-Serag HB, Rudolph KL (2007). Hepatocellular carcinoma: epidemiology and molecular carcinogenesis. Gastroenterology.

[2] Schwartz M (2004). Liver transplantation for hepatocellular carcinoma. Gastroenterology.

[3] Llovet JM, Ricci S, Mazzaferro V, Hilgard P, Gane E, Blanc JF, de Oliveira AC, Santoro A, Raoul JL, Forner A, Schwartz M, Porta C, Zeuzem S (2008). Sorafenib in advanced hepatocellular carcinoma. N Engl J Med.

[4] Jonker JW, Wagenaar E, Van Eijl S, Schinkel AH (2003). Deficiency in the organic cation transporters 1 and 2 (Oct1/Oct2 [Slc22a1/Slc22a2]) in mice abolishes renal secretion of organic cations. Mol Cell Biol.

[5] Zwart R, Verhaagh S, Buitelaar M, Popp-Snijders C, Barlow DP (2001). Impaired activity of the extraneuronal monoamine transporter system known as uptake-2 in Orct3/Slc22a3-deficient mice. Mol Cell Biol.

[6] Bachmakov I, Glaeser H, Endress B, Morl F, Konig J, Fromm MF (2009). Interaction of beta-blockers with the renal uptake transporter OCT2. Diabetes Obes Metab.

[7] Breidert T, Spitzenberger F, Grundemann D, Schomig E (1998). Catecholamine transport by the organic cation transporter type 1 (OCT1). Br J Pharmacol.

[8] Dudley AJ, Bleasby K, Brown CD (2000). The organic cation transporter OCT2 mediates the uptake of beta-adrenoceptor antagonists across the apical membrane of renal LLC-PK(1) cell monolayers. Br J Pharmacol.

[9] Grundemann D, Koster S, Kiefer N, Breidert T, Engelhardt M, Spitzenberger F, Obermuller N, Schomig E (1998). Transport of monoamine transmitters by the organic cation transporter type 2, OCT2. J Biol Chem.

[10] Grundemann D, Schechinger B, Rappold GA, Schomig E (1998). Molecular identification of the corticosterone-sensitive extraneuronal catecholamine transporter. Nat Neurosci.

[11] Jonker JW, Schinkel AH (2004). Pharmacological and physiological functions of the polyspecific organic cation transporters: OCT1, 2, and 3 (SLC22A1-3). J Pharmacol Exp Ther.

[12] Shikata E, Yamamoto R, Takane H, Shigemasa C, Ikeda T, Otsubo K, Ieiri I (2007). Human organic cation transporter (OCT1 and OCT2) gene polymorphisms and therapeutic effects of metformin. J Hum Genet.

[13] Thomas J, Wang L, Clark RE, Pirmohamed M (2004). Active transport of imatinib into and out of cells: implications for drug resistance. Blood.

[14] Zhang S, Lovejoy KS, Shima JE, Lagpacan LL, Shu Y, Lapuk A, Chen Y, Komori T, Gray JW, Chen X, Lippard SJ, Giacomini KM (2006). Organic cation transporters are determinants of oxaliplatin cytotoxicity. Cancer Res.

[15] Heise M, Lautem A, Knapstein J, Schattenberg JM, Hoppe-Lotichius M, Foltys D, Weiler N, Zimmermann A, Schad A, Grundemann D, Otto G, Galle PR, Schuchmann M (2012). Downregulation of organic cation transporters OCT1 (SLC22A1) and OCT3 (SLC22A3) in human hepatocellular carcinoma and their prognostic significance. BMC Cancer.

[16] Lautem A, Heise M, Grasel A, Hoppe-Lotichius M, Weiler N, Foltys D, Knapstein J, Schattenberg JM, Schad A, Zimmermann A, Otto G, Lang H, Galle PR (2013). Downregulation of organic cation transporter 1 (SLC22A1) is associated with tumor progression and reduced patient survival in human cholangiocellular carcinoma. Int J Oncol.

[17] Hyrsova L, Smutny T, Trejtnar F, Pavek P (2016). Expression of organic cation transporter 1 (OCT1): unique patterns of indirect regulation by nuclear receptors and hepatospecific gene regulation. Drug Metab Rev.

[18] Chen L, Shu Y, Liang X, Chen EC, Yee SW, Zur AA, Li S, Xu L, Keshari KR, Lin MJ, Chien HC, Zhang Y, Morrissey KM (2014). OCT1 is a high-capacity thiamine transporter that regulates hepatic steatosis and is a target of metformin. Proc Natl Acad Sci USA.

[19] Miller DM, Thomas SD, Islam A, Muench D, Sedoris K (2012). c-Myc and cancer metabolism. Clin Cancer Res.

[20] Kang KW, Im YB, Go WJ, Han HK (2009). C-myc amplification altered the gene expression of ABC- and SLC-transporters in human breast epithelial cells. Mol Pharm.

[21] Hilgendorf C, Ahlin G, Seithel A, Artursson P, Ungell AL, Karlsson J (2007). Expression of thirty-six drug transporter genes in human intestine, liver, kidney, and organotypic cell lines. Drug Metab Dispos.

[22] Crossman LC, Druker BJ, Deininger MW, Pirmohamed M, Wang L, Clark RE (2005). hOCT 1 and resistance to imatinib. Blood.

[23] White DL, Saunders VA, Dang P, Engler J, Venables A, Zrim S, Zannettino A, Lynch K, Manley PW, Hughes T (2007). Most CML patients who have a suboptimal response to imatinib have low OCT-1 activity: higher doses of imatinib may overcome the negative impact of low OCT-1 activity. Blood.

[24] White DL, Saunders VA, Dang P, Engler J, Zannettino AC, Cambareri AC, Quinn SR, Manley PW, Hughes TP (2006). OCT-1-mediated influx is a key determinant of the intracellular uptake of imatinib but not nilotinib (AMN107): reduced OCT-1 activity is the cause of low in vitro sensitivity to imatinib. Blood.

[25] Herraez E, Lozano E, Macias RI, Vaquero J, Bujanda L, Banales JM, Marin JJ, Briz O (2013). Expression of SLC22A1 variants may affect the response of hepatocellular carcinoma and cholangiocarcinoma to sorafenib. Hepatology.

[26] Grimm D, Lieb J, Weyer V, Vollmar J, Darstein F, Lautem A, Hoppe-Lotichius M, Koch S, Schad A, Schattenberg JM, Worns MA, Weinmann A, Galle PR (2016). Organic cation transporter 1 (OCT1) mRNA expression in hepatocellular carcinoma as a biomarker for sorafenib treatment. BMC Cancer.

[27] Cui R, Okada Y, Jang SG, Ku JL, Park JG, Kamatani Y, Hosono N, Tsunoda T, Kumar V, Tanikawa C, Kamatani N, Yamada R, Kubo M (2011). Common variant in 6q26-q27 is associated with distal colon cancer in an Asian population. Gut.

[28] Park T, Yi SG, Shin YK, Lee S (2006). Combining multiple microarrays in the presence of controlling variables. Bioinformatics.

[29] Staal YC, van Herwijnen MH, van Schooten FJ, van Delft JH (2006). Modulation of gene expression and DNA adduct formation in HepG2 cells by polycyclic aromatic hydrocarbons with different carcinogenic potencies. Carcinogenesis.

[30] Le Vee M, Jouan E, Stieger B, Fardel O (2013). Differential regulation of drug transporter expression by all-trans retinoic acid in hepatoma HepaRG cells and human hepatocytes. Eur J Pharm Sci.

[31] Fu L, Qin YR, Ming XY, Zuo XB, Diao YW, Zhang LY, Ai J, Liu BL, Huang TX, Cao TT, Tan BB, Xiang D, Zeng CM (2017). RNA editing of SLC22A3 drives early tumor invasion and metastasis in familial esophageal cancer. Proc Natl Acad Sci USA.

[32] Chen F, Beezhold K, Castranova V (2009). JNK1, a potential therapeutic target for hepatocellular carcinoma. Biochim Biophys Acta.

[33] Davis RJ (2000). Signal transduction by the JNK group of MAP kinases. Cell.

[34] Chang L, Karin M (2001). Mammalian MAP kinase signalling cascades. Nature.

[35] Chang Q, Chen J, Beezhold KJ, Castranova V, Shi X, Chen F (2009). JNK1 activation predicts the prognostic outcome of the human hepatocellular carcinoma. Mol Cancer.

[36] Hui L, Zatloukal K, Scheuch H, Stepniak E, Wagner EF (2008). Proliferation of human HCC cells and chemically induced mouse liver cancers requires JNK1-dependent p21 downregulation. J Clin Invest.

[37] Li WC, Ralphs KL, Tosh D (2010). Isolation and culture of adult mouse hepatocytes. Methods Mol Biol.

[38] Bradford MM (1976). A rapid and sensitive method for the quantitation of microgram quantities of protein utilizing the principle of protein-dye binding. Anal Biochem.

[39] Tamano S, Merlino GT, Ward JM (1994). Rapid development of hepatic tumors in transforming growth factor alpha transgenic mice associated with increased cell proliferation in precancerous hepatocellular lesions initiated by N-nitrosodiethylamine and promoted by phenobarbital. Carcinogenesis.

[40] Arndt P, Volk C, Gorboulev V, Budiman T, Popp C, Ulzheimer-Teuber I, Akhoundova A, Koppatz S, Bamberg E, Nagel G, Koepsell H (2001). Interaction of cations, anions, and weak base quinine with rat renal cation transporter rOCT2 compared with rOCT1. Am J Physiol Renal Physiol.

[41] Muller J, Lips KS, Metzner L, Neubert RH, Koepsell H, Brandsch M (2005). Drug specificity and intestinal membrane localization of human organic cation transporters (OCT). Biochem Pharmacol.

