# Supplementary material for: Loss of organic cation transporter 3 (Oct3) leads to enhanced proliferation and hepatocarcinogenesis
Source: Oncotarget. 2017 Dec 18;8(70):115667–80. doi: 10.18632/oncotarget.23372 (PMC5777802; doi:10.18632/oncotarget.23372)
Supplement: Supplementary file 1 [file oncotarget-08-115667-s001.pdf]

Loss of organic cation transporter 3 (Oct3) leads to enhanced proliferation and hepatocarcinogenesis

SUPPLEMENTARY MATERIALS

Supplementary Table 1: Liver enzymes.

	WT Control (n=5)	Oct3$^{-/-}$ Control (n=6)	p-value	WT DEN/Phen (n=6)	Oct3$^{-/-}$ DEN/Phen (n=12)	p-value
ALT	45.2 (±18.7)	53.2 (±26.3)	0.62	50.4 (±7.3)	121.1 (±97.8)	0.16
AST	66 (±48.9)	119.7 (±67.1)	0.61	118.2 (±31.3)	189.8 (±74.2)	0.08
AP	46 (±7.5)	51.7 (±7.0)	0.71	55.2 (±5.6)	84.4 (±30.5)	0.07
Total bilirubin	<0.1	0.1 (±0.02)	0.39	0.1 (±0)	0.2 (±0.1)	0.42
LDH	268 (±24.2)	432.3 (±238.9)	0.29	712 (±191.8)	751.6 (±308.4)	0.81

No significant differences were found for activity of serum alanine transaminase (ALT) and aspartate transaminase (AST), alkaline phosphatase (AP), bilirubin and lactate dehydrogenase (LDH) between 10 month old untreated and DEN/Phenobarbital (Phen) treated Oct3$^{-/-}$ and WT mice.

Supplementary Table 2: Oligonucleotides

Transcript	Length	Direction	Primer
Slc22A3	115 bp		HS_SLC220A3_1_SC
Ki-67	20 bp	forward	5’-TCT GAT GTT AGG TGT TTG AG-3’
	21bp	reverse	5’-CAC TTT TCT GGT AAC TTC TTG-3’
CyclinD1	20 bp	forward	5’-CAA TGA CCC CGC ACG ATT TC-3’
	19 bp	reverse	5’-CAT GGA GGG CGG ATT GGA A-3’

Primers applied for qPCR.

Supplementary Table 3: Antibodies

Protein	Antibody
AKT	rabbit-anti-AKT monoclonal antibody (1:1000, Cell Signaling Technology, Inc, Cambridge, UK)
P-AKT	rabbit-anti-P-AKT monoclonal antibody (1:1000, Cell Signaling Technology, Inc, Cambridge, UK)
MEK	rabbit-anti-MEK1/2 monoclonal antibody (1:1000, Cell Signaling Technology, Inc, Cambridge, UK)
P-MEK	rabbit-anti-P-MEK1/2 monoclonal antibody (1:1000, Cell Signaling Technology, Inc, Cambridge, UK)
AMPKβ	rabbit-anti-AMPK-b monoclonal antibody (1:1000, Cell Signaling Technology, Inc, Cambridge, UK)
P-AMPKβ	rabbit-anti-P-AMPK-b monoclonal antibody (1:1000, Cell Signaling Technology, Inc, Cambridge, UK)
AMPKα	rabbit-anti-AMPK-a monoclonal antibody (1:1000, Cell Signaling Technology, Inc, Cambridge, UK)
P-AMPKα	rabbit-anti-P-AMPK-a monoclonal antibody (1:1000, Cell Signaling Technology, Inc, Cambridge, UK)
ERK	rabbit-anti-MAPK (Erk1/2) monoclonal antibody (1:1000, Cell Signaling Technology, Inc, Cambridge, UK)
P-ERK	rabbit-anti-P-MAPK (Erk1/2) monoclonal antibody (1:1000, Cell Signaling Technology, Inc, Cambridge, UK)
JNK	rabbit-anti-SAPK/JNK monoclonal antibody (1:1000, Cell Signaling Technology, Inc, Cambridge, UK)
P-JNK	rabbit-anti-P-SAPK/JNK monoclonal antibody (1:1000, Cell Signaling Technology, Inc, Cambridge, UK)

Antibodies applied for Western Blotting.
